# Individualizing the burnout problem: Health professionals’ discourses of burnout and recovery in the context of rehabilitation

**DOI:** 10.1177/13634593211063053

**Published:** 2021-12-02

**Authors:** Maija Korhonen, Katri Komulainen

**Affiliations:** University of Eastern Finland, Finland

**Keywords:** burnout rehabilitation, contested forms of mental distress, discourse analysis, categorization, health professionals

## Abstract

This discourse analytical study explores how health professionals (HPs) construct burnout as a form of mental distress in the context of Finnish burnout rehabilitation framed with a particular rehabilitation ethos. Burnout is a fuzzy concept and lacks a disease status. Therefore, it calls for context-specific definition and justification. By highlighting the socially and interactionally produced character of categories of mental distress, the study investigates the kinds of discourses HPs use to formulate “the problem” and its solutions, and how people dealing with burnout are categorized in these discourses. The data consists of field notes from the observation of group discussion sessions in two 1-year burnout rehabilitation courses. As a result of the analysis, five partly overlapping discourses were identified: psychological, evolutionary, healthy lifestyle, biomedical, and welfare. Within these discourses, people who experience burnout were categorized as over-conscientious employees, “good girls,” “primitive people,” self-responsible rehabilitees, patients, and (aging) employees with social and legal rights. Burnout rehabilitation and HPs’ views reproduce a cultural and clinical discourse around burnout in which work-related problems are treated as individual-level problems and individuals are responsibilized for the management of mental distress. Based on the results, it is concluded that the hybrid type of interventions that attempt to influence both individual- and work-related problems behind burnout would help to prevent people dealing with burnout from being over-responsibilized for solving problems at the workplace.

## Introduction: Discourse analytic approach to mental distress

This discourse analytic study is concerned with exploring how health professionals (HPs) construct burnout as a form of mental distress in the institutional setting of rehabilitation. Drawing on the field notes from the observation of group discussions in two Finnish group-based burnout rehabilitation courses, we analyse the kinds of discourses with certain categorizations of people dealing with burnout that are constructed by HPs. In previous discourse analytical (DA) research on mental distress, the focus of the analysis has often been on the ways by which individual patients react to receiving a diagnosis and construct “diagnostic identities” in light of such a diagnosis (Tucker, 2009). This study aims to shift the emphasis from a psychological process to interpersonal and interactional processes and culturally available discourses that frame and shape the understanding of distress (Georgaca, 2013). Following the DA studies of professionals’ accounts, the study highlights the socially produced character of categories of mental distress and the ways by which professionals use these categories to attribute responsibility, claim professional expertise and justify professional practices (Georgaca, 2013).

The starting point of this study is, first, that the way mental distress is discursively constructed in institutional contexts is crucial to the positions that become available to clients—and thereby the type of support they may encounter (Ringer and Holen, 2016: 162). However, there is a paucity of DA work on professional practices in institutional settings (Georgaca, 2013), such as rehabilitation. Second, we suggest that one of the functions of any kind of rehabilitation is to define and establish what the specific problem at issue is, so that a problem can be worked through. Problems are not objective things waiting to be detected, diagnosed, and treated; rather, they are constituted in discursive practices (Savic et al., 2017: 80). The need to define the problem is emphasized in the case of mental distress that does not meet the diagnostic criteria for disease and, therefore, lacks a specific explanatory framework (Korhonen and Komulainen, 2018).

In this study, we approach burnout as such a form of mental distress which, due to its lack of disease status, requires context-specific definition and justification. Even though burnout has been a major epidemic in several Western and developing countries since the early 2000s (Eurofound, 2018; Maslach et al., 2001), scientists and practitioners are still debating what burnout actually is (Bianchi et al., 2015; Heinemann and Heinemann, 2017a). Because of its conceptual fuzziness and symptomatic overlap with depression and anxiety, burnout is not officially recognized as a distinct mental disorder in most countries, including Finland^1^ (Heinemann and Heinemann, 2017a). When it comes to contested disorders or forms of mental distress such as burnout, also subsequent treatment becomes questionable (Bech Risor and Lillevoll, 2021).

Drawing on the above notions, we ask how burnout as a problem is constructed and how recovery is viewed by HPs in the context of burnout rehabilitation. What kinds of discourses do HPs use to formulate, define, and explain the problem and its solutions, and how are people who experience burnout portrayed and categorized in these discourses? Based on our analysis, we reflect on whether—and in which ways—the discourses applied by HPs resonate with the ethos of responsibilization embedded in health care policy and practices (Raitakari et al., 2019). To our knowledge, this is the first study to investigate the construction of burnout and the implementation of burnout intervention from a DA point of view. The study offers perspectives for both scholars and practitioners to critically reflect on professional practices around non-diagnostic forms of mental distress and the discursive effects of these practices (see Georgaca, 2013).

## Understanding the burnout problem

### Academic and practice-based approaches to burnout

According to Schaufeli (2017: 105), the term “burnout” was first used in a clinical sense and in the public sector work in the early 1970s by Herbert Freudenberger, a practicing American psychologist who applied the term to describe the gradual emotional exhaustion he witnessed among volunteers in the drug clinic. In the 1980s, the concept was further developed as the scientific research around burnout began to grow. From the beginning, there have been at least two more or less independent streams in the field of burnout studies: (1) an academic research and (2) a practice-based approach focused on burnout interventions (Schaufeli, 2017).

Within academic research,^2^ the long tradition of causal theorizing has focused on both situational and individual factors that cause people to experience burnout (Maslach and Leiter, 2016). As Maslach and Leiter (2016) summarize this line of research, situational factors or problems causing burnout include, for example, work overload, a lack of control over one’s work, insufficient recognition and reward and a lack of support, trust and respect at the workplace. Individual causes behind burnout have been traced to personality characteristics (such as neuroticism, perfectionism, low levels of hardiness), work-related attitudes (such as idealistic expectations toward work) and coping styles (Maslach et al., 2001). However, there is a relative consensus among researchers that *job burnout*^3^ is not a problem of individuals but of the social environment in which they work (Maslach and Leiter, 2017: 160).

Nevertheless, especially practice-based research and clinical practice are based on the psychological research framework (Maslach, 2017) and emphasize the contribution of individual factors to the development of burnout (Hakanen, 2018; Schaufeli and Enzmann, 1998). In the ICD-11, burnout (code Z73.0) is placed in the category “problems related to life management difficulty.” This definition introduces burnout as an individual, not work-related problem (Eurofound, 2018).

Maslach (2017) characterizes several implications that result from the psychological, individual-centered definition of burnout. First, such an approach embodies the Western individualistic ideology, which celebrates the triumph of the individual over any obstacle (see also Leiter and Maslach, 2014). Thus, the person is regarded as responsible for doing something about the burnout problem. Second, burnout is easily stigmatized as a sign of weakness, incompetence or even a psychological “disease” at the workplace. Finally, the message that is often implicitly (if not explicitly) conveyed by individual-focused understanding of burnout is one of blaming the victim (Maslach, 2017; Mueller and Morley, 2020).

The individual-centered understanding of burnout has also occurred in public discussion. In the Finnish context, burnout has been treated as a problem with an individual’s working ability rather than as a problem resulting from social or structural problems in working life (Rikala, 2013). It should be noted, however, that lay understanding of burnout varies between cultural contexts (Heinemann and Heinemann, 2017b) and may also be in contrast to the above discussed individual-centered discourse so that burnout is considered a normal psychological response to an abnormal work-related pressure (Schaufeli, 2017) or as a social problem related to neoliberalization of society and working life (Heinemann and Heinemann, 2017b).

### Individual-oriented burnout interventions and the ethos of responsibilization in rehabilitation

Perceptions of the prevention and treatment of burnout vary from those who highlight the individual’s responsibilities to those who call for organization-level actions (Hakanen, 2018). So far most of the burnout interventions have been person-oriented aiming to fix the person rather than the job (Awa et al., 2010; Maslach and Leiter, 2017). Job-oriented or organization-directed interventions which try to change the work conditions are relatively rare despite the research evidence for the primary role of situational factors behind burnout (Maslach and Leiter, 2017).

This is also the case in the Finland where the main goal of rehabilitation targeted at people who have problems with their working ability has been to equip individuals to manage stress (Järvikoski, 2013). This focus can be explained by the wider change in the ideals and practices of Finnish rehabilitation—namely the shift from a disease-based and medical frame of reference to a psychosocial framework^4^ and discourse. According to this framework, the reinforcement of an individual’s agency, empowerment, coping and a sense of control over their own wellbeing is a central objective in Finnish rehabilitation (Hätinen, 2008). Individuals’ agency and wellbeing are reinforced, for example, by helping the person to change their attitudes and work patterns and to improve their self-understanding via self-analytic techniques such as mindfulness (Ahola et al., 2017; see also Maslach and Leiter, 2017).

In line with the Nordic welfare model, state-funded rehabilitation in Finland has been seen as a part of social protection. However, the welfare model in Finland as well as in the other Nordic countries is changing rapidly. The neoliberal policy has increasingly placed the role of markets at the center of economic and social life, and there has been a shift in the ethos of the welfare model from universalism, solidarity and equity toward individualism and efficiency (Marjanen et al., 2018). Researchers have used such notions as “active citizenship” or “responsibilisation” (Raitakari et al., 2019) to conceptualize the neoliberal governance that is grounded upon the self-activating capacities of free subjects.

In the Australian context Mueller and Morley (2020) argue that the dominant discourse surrounding workplace stress further promotes individual responsibilization by blaming the individual experiencing burnout and obscuring the role of the organizational context in it. Mueller and Morley (2020) further suggest that current victim blaming understanding of burnout is an individualized neoliberal construction. The crucial thing, then, is to reflect on whether the Finnish burnout rehabilitation (re)produces such a discourse around burnout in which work-related problems become individualized in a neoliberal fashion.

## Study design

### Research setting: Burnout rehabilitation courses

The setting of the study consists of two Finnish group-based burnout rehabilitation courses^5^ (later groups A and B). The study is part of a larger follow-up research project, which was conducted in 2016–2018. Both courses, funded by the Social Insurance Institution of Finland (Kela), lasted about 1 year and included three 5-day group-based rehabilitation periods at the rehabilitation center. The rehabilitation was targeted at employees whose working ability was already jeopardized by symptoms of burnout (vs preventive). Its official aim was to enhance and maintain the participants’ well-being at work and acknowledge the significance of a healthy lifestyle and the importance of active self-responsibility for recovery.

Each 5-day rehabilitation period in both courses included a total of 22–24 hours of group discussion sessions guided by different HPs (see Table 1). The investigated rehabilitation courses were identical in terms of their general aims and contents, although the daily scheduled meetings and thematic contents of the group discussions varied somewhat between the two courses. In addition to the group discussions, the group-based programs involved joint exercise, relaxation exercises and nature trips. Moreover, the rehabilitees had individual meetings with different HPs.

**Table 1.** Overview of discourses and categories.

Timing in rehabilitation	Theme of group discussion session	HP	Discourse(s)	Formulation of the problem	Formulation of the solution	Categorization of people with burnout• Category-based features and actions
1. Rehabilitation period	Personal burnout narratives(groups A + B) • Sharing personal burnout narratives	Psychologist, physiotherapist	Life-historical Psychological	Certain psychological characteristics lead to burnout Changes and difficulties in working life lead to burnout	Recovery requires learning assertiveness	Lifelong good employee • Hard-working, diligent, conscientious Over-conscientious employee • Demanding toward her-/himself, over-performing
	Causes and consequences of burnout (groups A + B) • Listing causes and consequences of burnout	Psychologist	Psychological	Certain psychological characteristics lead to burnout	Recovery requires learning assertiveness	Over-conscientious employee “Good girl” • Bearer of responsibilities, puts other people’s needs ahead of her own well-being
	Medical treatment of burnout (groups A + B) • Lecture about symptoms and treatment of burnout and depression	Psychiatrist	Biomedical Healthy lifestyle Evolutionary	Un(der)treated burnout leads to illness, that is depression	Recovery requires healthy lifestyle and medical and psychological treatment	Patient • Suffers from symptoms that require medical and/or psychological treatment Self-responsible rehabilitee • Bears individual responsibility for the self-care of the mind and body Primitive people • Acts on the basis of his/her biological nature
	Personal rehabilitation goals (groups A + B) • Sharing and evaluating personal rehabilitation goals	Psychologist	Healthy lifestyle Psychological	–	Recovery requires healthy lifestyle and learning assertiveness	Self-responsible rehabilitee Over-conscientious employee ”Good girl”
2. Rehabilitation period	Body awareness and mindfulness (group A) • Lecture, discussion and mindfulness exercises	Occupational therapist	Evolutionary Biomedical Healthy lifestyle	Modern way of (working) life prevents us from dealing with stress in a natural way	Recovery requires slowing down and healthy lifestyle	Primitive people Patient Self-responsible rehabilitee
	Personal work histories (groups A + B) • Sharing work histories	Psychologist	Life-historical	Certain psychological characteristics lead to burnout Changes and difficulties in working life lead to burnout	Recovery requires learning assertiveness	Lifelong good employee
	Options for lightening work (group A) • Lecture about statutory options to support work ability	Social worker	Welfare	–	Recovery requires lightening workload	(Aging) employee with social and legal rights Employee with reduced work ability
	Stress and stress management (groups A + B) • Listing “good” and “bad” stress and means of stress management	Psychologist	Healthy lifestyle	–	Recovery requires healthy lifestyle	Self-responsible rehabilitee
	Sleep and insomnia (group B) • Discussion about sleeping habits and insomnia	Psychiatric nurse	Psychoanalytic (Jungian)	–	–	–
	Means of self-care for mental health (groups A + B) • HCP listing the means of self-care	Psychotherapist	Evolutionary Healthy lifestyle Psychological Biomedical	–	Recovery requires slowing down, healthy lifestyle, and learning assertiveness	Primitive people Self-responsible rehabilitee “Good girl”
	Coping, mood and life satisfaction (group B) • Evaluating one’s coping, mood and life satisfaction on a scale of 0–10.	Psychotherapist	Healthy lifestyle Psychological	–	Recovery requires healthy lifestyle and learning assertiveness	Over-conscientious employee
3. Rehabilitation period	Coping resources (group B) • Drawing pictures about resources related to childhood memories	Psychiatric nurse	Psychoanalytic (Jungian)	–	–	–
	Sleep and memory (group A) • Lecture	Occupational therapist	Evolutionary Healthy lifestyle	–	Recovery requires slowing down and healthy lifestyle	Primitive people Self-responsible rehabilitee
	Orientation toward the future (groups A + B) • Rehabilitation experiences, future goals for better well-being	Psychologist + physiotherapist	Healthy lifestyle Psychological	–	Recovery requires healthy lifestyle and learning assertiveness	Self-responsible rehabilitee Over-conscientious employee

Both the rehabilitation participants and HPs involved in the study were recruited in cooperation with the rehabilitation center and the responsible actors for the chosen courses (a psychologist and two physiotherapists). Research permission was obtained from other HPs with the help of these key actors, who also forwarded the research information letter to the clients prior to the beginning of the rehabilitation. In the information letter, participants were briefed on the aims and venue of the study, voluntary participation, their right to withdraw from the study at any stage, data usage, processing and archiving, and confidentiality. Moreover, the letter highlighted that participation in the study or refusal to participate in it did not affect the implementation of the rehabilitation program. Ethical approval for the research was given by the University of Eastern Finland Committee on Research Ethics.

### Data: Observation and field notes

The data used in this study—field notes gathered by means of observations of group discussion sessions on both rehabilitation courses—is drawn from data from a larger project, which also consists of follow-up interviews with rehabilitation participants. Altogether 19 people (18 women and one man) participated in two courses. All of them granted permission for the observation of the group discussions, and 15 (14 women and one man) participated in follow-up interviews.

The participants were 34–62 years old (at the beginning of rehabilitation) and represented various occupations.^6^ They worked in the fields of health care and social work, technology, education, media, and the service sector. Most of the participants were highly educated. They had had a relatively long and stable career and they perceived themselves as qualified employees. Subjectively reported in the interviews, heavy workloads, long hours, a pressured work environment and poor management had determined the participants’ situation.

The observation data comprises a total of 324 pages (A4) of hand-written field notes, some of which were later transferred to a computer for analytical purposes. The observation was conducted by the first author. During the group sessions, the researcher avoided any comments in the discussions, as was agreed in advance with the rehabilitation staff. The researcher’s non-involvement in discussion made it easier for her to hear different voices and minimize the effect she had on the setting and the people being studied. Because it was impossible to collect data on everything that was said and that was going on, the researcher needed, by necessity, to be selective in her writing (Eriksson et al., 2012). It should be noted, too, that the field notes are reductions of naturally occurring discussions. The writing of field notes was guided by a few theoretical-methodological assumptions, which filtered the data being gathered and its interpretation.

First, according to the general principles of critical health psychology, rehabilitation practices were not regarded as exclusively emancipatory, but also as having normative functions (e.g. Juhila et al., 2016). Second, it was assumed that discursive meaning making involves multiple “voices,” some of which may be dominant while others remain in the margins (Korhonen and Komulainen, 2017). Therefore, the researcher observed the situations mainly from the point of view of what was being said (vs group interaction, group dynamics, and non-verbal communication) and how the voices of the speakers varied.^7^ She also made notes with a more reflective or analytical tone. The researcher had previously worked as a psychologist on similar rehabilitation courses. Her prior experience partly guided (and possibly restricted) the ways she viewed, understood, and mapped out talk by HPs (Berger, 2015). However, familiarity with the context also enabled better in-depth understanding of what was being said.

### Method: Discourse analysis and categorization

The data was analyzed from the DA and categorization point of view. Our approach was guided by some generally accepted assumptions about the relationships between discourse and social processes. First, we use DA as a method of examining the function of socially determined systematic ways of speaking; our DA entails studying language use as a form of social practice which constructs versions of the social order (Georgaca, 2013).

Second, we suggest that discourses as social practices frame the way individuals and groups conceptualize themselves and others. In addition to DA, we apply an ethnomethodologically oriented categorization analysis to analyse in detail how HPs display and use discourses to create constructions of people dealing with burnout. As Juhila and Abrams (2011) argue, categorization, is an inherent part of the interaction between individuals, and in professional talk categories are often used in specific institutional tasks. According to Juhila et al. (2010), categories store cultural knowledge and the processes of categorization work as sense-making devices in the reasoning of health and illness. When people are categorized in interaction, these categories invoke assumptions about particular features and actions bound to them (Sacks, 1992). Placing people in categories is consequential because categories produce social identities and the related moral expectations for people (Juhila and Abrams, 2011). Moreover, categorizing people, especially related to health and illness, is closely tied with the notions of normality and deviance (Korhonen and Komulainen, 2018).

Although discourses used by HPs occur in group discussions with rehabilitees and are, therefore, relationally constructed, observational field notes do not allow for a detailed analysis of the interaction. Rather than exploring the negotiation processes taking place between HPs and clients, we are interested in what kinds of categories of people dealing with burnout HPs create in their discursive practices (Georgaca, 2013) while they strive to explain burnout as a problem and identify means of recovery. Categories are not necessarily directly named by the HPs; instead, certain ways of talking about course participants discursively produce categories which we, as researchers, have sought to crystallize by naming them.

Our analysis proceeded with the following steps. First, the first author conducted an in-depth reading of the whole observation data. She explored the themes of group discussions held by different HPs and identified all the speech episodes in which the problem and solutions to the problem were represented by HPs (see Savic et al., 2017). Second, she looked at what exactly was regarded as the problem, what assumptions underlied the representation of the problem, and what discursive effects were produced by this representation of the problem. Third, she identified the explanatory frameworks through which the representations of the problem were articulated and looked at the ways in which people who experience burnout were categorized in these frameworks. Based on this, she outlined the preliminary discourses and categories that prevailed systematically and repeatedly in the data. This included comparison of similarities and differences between discourses and categories. In the fourth phase, preliminary discourses and categories and the related interpretations were discussed in joint data sessions with the second author, and some of the previously identified discourses and categories were refined and partly modified.

## Results

### Overview of discourses and categories

Table 1 represents the topics of group discussions and an overview of the discourses and categorizations through which burnout and recovery were constructed by the HPs in different thematic group discussions. The identified discourses embodied different perspectives and nuances of the problem formulation and the related categorizations. In practice, the discourses and categorizations overlapped, and several discourses could be drawn on simultaneously by HPs during the same group discussion. Some of the group discussions focused mainly on defining the problem and its causes, whereas others aimed at identifying solutions to the problem.

Next, we illustrate the variation of discourses and categorizations by taking a closer look at the discursive practices applied by HPs.

### Psychological discourse

The psychological discourse occurred particularly in one group discussion in which the HP introduced a popular psychology book entitled *Self-Treatment of Burnout.*^8^ The title of the book suggests that recovery from burnout requires self-care. However, the metaphorical notion derived from the book that “burnout is setting up camp at a crossroads in life” served as an instructional tool in encouraging the participants’ reflection on the causes and outcomes of their burnout:

HP writes the sentence “burnout is setting up camp at a crossroads in life” on the flip chart and draws a tree with roots and forking branches. Talks about the factors that may have led to “the crossroads” and writes these on the flip chart: “1) habits, 2) personality traits, 3) things beyond your control, circumstances.” Refers to studies: “The typical profile of someone with burnout is a kind woman with difficulties drawing boundaries, saying no, standing up for her rights.” Instructs every rehabilitee to draw “the crossroads of their own life” on paper. [Field notes, Causes and consequences of burnout, group A, 25 August 2016.]^9^

After instructing the participants to consider the crossroads of their lives, the HP started to speak about “traits” and “personality” that are characteristic of each “path,” thereby placing the roots of burnout within the individual. HP gave a short list of personality characteristics, such as “niceness” and “inability to set boundaries,” “softness,” and “consuming professional ambition,” which were construed as problematic from a well-being point of view. Thus, the idea of burnout as a crossroads worked to construct a view concerning an individual-level problem and acted as an invitation for the participants to observe and reflect on their psychological characteristics, in particular.

Within this discourse, people dealing with burnout were categorized as *over-conscientious employees* whose certain psychological characteristics had made them vulnerable to burnout. Moreover, the psychological discourse produced a gendered categorization of burnout women as “*good girls*” who have a tendency to take excessive responsibility and care for other people and sacrifice their own needs. Through this categorization, many of the participants’ feminine-assumed psychological characteristics and behavioral patterns appeared deviant and, therefore, in need of change. Accordingly, recovery from burnout was seen to require learning to become “merciful” to oneself and learning to become more “assertive” because circumstances at work “cannot be influenced”:

HP notes that “burnout leads to depression” and suggests that all of the rehabilitees “should give yourselves time to heal, you have to be merciful.” Speaks about “learning assertiveness.” Says that assertiveness “is being firm, and sometimes you have to upset someone else, when you set boundaries.” Continues that “there’s nothing wrong with being nice, but martyrdom, self-sacrifice is its opposite.” [Field notes, Coping, mood and life satisfaction, group B, 6 March 2017.]

HP says that “since you can’t influence your work, it’s important to learn how to identify the things that you can affect with your thinking.” Continues that “taking care of yourself is the greatest form of selflessness” and that ”it’s important to set boundaries in life in general”. [Field notes, Means of self-care for mental health, group A, 26 August 2016.]

### Evolutionary, biomedical, and healthy lifestyle discourses

Burnout and recovery were also interpreted through the evolutionary discourse that occurred in several group discussions led by different HPs. Firstly, evolutionary ideas were taken up as a way of constructing the *causes* of burnout: stress was seen as a result of a discordance between humans’ ancient, genetically determined biology and the activity patterns of contemporary people (see also Björklund and Wright, 2017). The problem was construed to indicate that modern (working) life prevents us from dealing with stress in a natural way and modern people are alienated from the messages of their minds and bodies, which potentially leads to burnout. People who experience burnout were categorized as *primitive people* who acted on the basis of their genetics:

HP talks about how [when you have burnout] your thoughts can revolve excessively around certain things. Says that “emphasising the negative, the negative bias, is an evolutionary phenomenon” and important “for the survival of the individual and the species.” Continues later by speaking that it is typical for humans that when “when a tight situation is over and intense action stops, your sympathetic nervous system kicks in.” Illustrates how primitive humans released stress with a comparison to animals. “That’s how nature intended it!.” Says that, in working life, “natural situations do not occur.” Continues that sometimes the “[stressful] situation goes on for years, months or you achieve an energetic state but there is nothing to release it on or you have to postpone the activity.” [Field notes, Body awareness and mindfulness, group A, 1 March 2017.]

Whereas in the psychological discourse burnout was constructed by pathologizing specific psychological characteristics, in the evolutionary discourse the mismatch between the biological characteristics of the human species and the characteristics of modern working life was in a way normalized: burnout seemed to be a “healthy” and normal reaction to external conditions into which individuals “drift.” Thus, changes in working life appeared inevitable and uncontrollable:

HP: “Modern humans have become estranged from the messages from their minds and bodies. We last spoke about how people are driven to burnout and depression without noticing it.” Continues later by saying that “burnout is a healthy reaction to impossible situations [in working life]. Working life has changed tremendously, but people have not.” [Field notes, Coping, mood and life satisfaction, group B, 6 March 2017.]

Second, the HPs construed views of *recovery* from burnout in the evolutionary discourse. Recovery was seen to require that burnout individuals—as “modern humans”—learn to carefully listen to the messages from their minds and bodies. Here, this discourse was intertwined with the biomedical and healthy lifestyle discourses.

As illustrated below, the biomedical discourse portrayed rehabilitees as *patients* with various symptoms that require medical and/or psychological treatment. The biomedical discourse constructed burnout as a legitimate “syndrome,” not as a deviance of an individual’s psychological character. Within the healthy lifestyle discourse, however, people dealing with burnout were categorized as *self-responsible rehabilitees* who should actively engage in managing their physical and mental well-being by means of self-care, such as a regular daily rhythm, meditation, and physical activity.

The psychiatrist utilized the biomedical, healthy lifestyle and evolutionary discourses to justify the benefits of medical and psychological treatment and a healthy lifestyle, especially physical activity, for recovery. HP presented a causal explanation according to which untreated burnout inevitably leads to a “syndrome” called depression and described the symptoms of depression, which were related to unhealthy, deviant habits (“sleeping and eating too much”) as well as undesired intra-psychological outcomes. Thus, the HP constructed the nature of the illness to be fought against:

HP says that “depression inevitably follows if the burnout persists.” Goes on by saying that the symptoms of depression include difficulties concentrating, a shortened attention span, lack of vigour and energy, self-reproach, and pessimism. HP says, “These are what make up depression as a syndrome, as opposed to a condition such as diabetes.” Later brings up depression medication and therapeutic support, discusses different types of medicines and talks about the neural basis of the medicines’ effects and the mechanism of action of neurotransmitters. Says that aerobic exercise has the same effect on neural networks as antidepressants: “Exercise is in itself neuroplastic. Humans are orienteers by nature.” [Field notes, Medical treatment of burnout, group B, 7 October 2016.]

The occupational therapist also drew on all three discourses while he encouraged rehabilitees to “take care of their brains” through healthy life habits and referred to an evolutionary perspective:

HP speaks of “resting the body” and “resting the brain.” Asks the group “What nutrient does the brain need to function?” Goes on to say that these are complemented by sleep and speaks of the “brain maintenance perspective.” Continues by saying that “humans are a day species” and that “daily routines are important from an evolutionary perspective.” Later also speaks of naps, meditation, and memory, referring to the evolution of the “human species,” for example the significance of memory to the “survival of the species.” Continues about the importance of exercise by saying: “It would be natural for humans to walk or run 20 km a day.” [Field notes, Sleep and memory, group A, 15 August 2017.]

In another group discussion, the psychotherapist highlighted the significance of physical exercise, rest, and relaxation for recovery by utilizing the biomedical and healthy lifestyle discourses. The psychotherapist talked about the “fight or flight-reaction” thereby referring to popular stress theories that regard the fight-or-flight response as our ancient response to stressful situations. By relying on the evolutionary discourse, HP suggested that social interaction with other people is a “species-specific need” for human beings:

HP: “Modern research has shown that just 10–15 minutes of light exercise has an effect comparable to antidepressants – you can tell yourself that you’re setting out to rehabilitate your brain.” Talks about the activation of the sympathetic nervous system and the fight or escape reaction to stressful situations. Provides instruction in relaxing the body and introduces mental self-care methods: “This is number one,” (writes the word “exercise” on the flip chart). Continues by talking about the effects of exercise on mood. Later lists other means of mental self-care, such as “rest,” “meaningful activities,” “taking a moment” and “idleness” (refers to “positive idleness” and stresses that “passivity is not empowering”), “relationships” (“our species has a need for connection with other human beings,” “a need at a cellular level”), “nourishment” and “nature experiences.” [Field notes, Means of self-care for mental health, group B, 8 March 2017.]

Moreover, HP pointed out the difference between the right kind of physical activity and “being passive” and construed an ideal of active self-management of mental health and performance, in particular, by emphasizing “rehabilitation of the brain.” Thus, the biomedical and healthy lifestyle discourses resonated with the neuro-discourse and related idea of self-optimization, which has become prevalent in workplace health interventions (e.g. Pykett and Enright, 2015).

In the following example, the psychotherapist utilizes the healthy lifestyle discourse and suggests the learning of rest and relaxation skills for the management of work stress. She denies the possibility of being able to affect the working conditions. The example illustrates that within the healthy lifestyle discourse, people who experience burnout were responsibilised for the control and management of their minds and bodies in the face of work-related difficulties.

Laura [one of the participants, Laura is a pseudonym] says that she went back to work for a week after her holiday and before this rehabilitation period. She says: “It was anything but smooth sailing!” Laura ironically recounts a newspaper’s tips on an easy start for work after the holidays. HP: “There’s nothing you can do about your work, but you can keep the feeling of not having enough time under control, so that it doesn’t get under your skin. That’s when it consumes your strength.” Talks about how “important it is to be aware whether you are giving yourself the opportunity for sufficient rest.” Later stresses the fact that “work doesn’t change.” [Field notes, Coping, mood and life satisfaction, group B, 8 August 2017.]

The group discussion held by a social worker was the only session in which recovery from burnout was approached from the point of view of work arrangements and the related solutions that could support the individual’s working ability. A social worker gave a lecture about social benefits, rehabilitation options, social security allowances and different statutory options for lightening workloads, such as applying for a part-time pension, disability pension, alternate leave, and part-time work. The excerpt below comes from part of that discussion:

Explains that there have been changes in the ways sick leave due to incapacity for work are addressed in the workplace, and this results from new legislation on health insurance and occupational health care. “When the employee’s illness is prolonged, the remaining work capacity must be assessed by an occupational health doctor. In addition, the employer, the employee, and the occupational health service work together to determine the employee’s chances of continuing to work.” [Field notes, Options for lightening work, group A, 17 August 2017.]

Within the *welfare discourse*, rehabilitees were categorized, first, as *employees with social and legal rights* who are entitled to collective, system- and organization-level support for their ability to work and, second, as *employees with reduced working ability.* Moreover, occupational health care and employers were responsibilized for finding solutions that support the employee’s working ability. In the welfare discourse, the participants were addressed especially as aging employees. This can be explained by the fact that in the Finnish social insurance system access to many social benefits to maintain working ability requires certain criteria related to the number of working years carried out and the age of the employee.

### Adopting and contesting individualized definitions

Although our focus in this study is on the ways HPs as institutional authorities constructed burnout and recovery, we also take a brief look at the rehabilitation participants’ own meaning making, within which they both adopted and challenged the discourses and categorizations created by the HPs.

The first group session of both rehabilitation courses focused on sharing personal burnout narratives. Within a *life-historical discourse*, participants constructed causal explanations for their burnout. They presented themselves as hardworking and conscientious employees, categorizing themselves as *lifelong good employees*. They stated things such as, “Work used to be my love, my friend, I devoted myself to work,” or, “I always worked long hours, diligent by nature, did what I was told,” [Field notes, Personal burnout narratives, group B, 3 October 2016].

The categorization of rehabilitees as lifelong good employees occurred also in the group sessions that dealt with personal work histories. Almost everyone emphasized hard work since childhood and the centrality of work in their lives: “All my life I have worked long hours,” and, “I was born in 1982, and that’s when I started to work,” [Field notes, Personal work histories, group A, 28 February 2017].

Although in both group sessions the participants perceived themselves as people for whom work had been the foundation of their identity, in the face of burnout their lifelong commitment to work became regarded as an individual flaw. In other words, the participants located the causes of burnout in themselves by characterizing themselves as over-conscientious “workaholics,” for example, and by presenting an ironic critique of the qualities of a good employee perceived in themselves. In this respect one of the participants stated: “It must be a mental disability then, stretching it out [work],” [Field notes, Personal burnout narratives, group B, 3 October 2016]. Thus, in the same vein as the HPs, the rehabilitees portrayed themselves as over-conscientious employees.

Whereas the HPs’ discursive practices emphasized individual-related causes of burnout, in their personal burnout stories and work histories the rehabilitees brought up adversities at work that they had faced at different times. They pointed out long-lasting and cumulative negative processes at work, such as increased workloads and poor working arrangements. They said: “No real breaks in my work” or, “Every year, we’ve had redundancy negotiations, people let go,” and, “Working life went off kilter after the depression” [Personal burnout narratives, group B, 3.10.2016]. However, the response from the HPs framed the discussion toward prioritizing individual attitudes and behaviors whilst downplaying the participants’ work-related concerns. For instance, one HP started to speak about the ability to “say no” in the face of difficult work situations and emphasized that solving problems at work was not a part of the aims of rehabilitation: “We may not be able to fully solve these problems here. Maybe it’s the person drawing the boundaries” (Field notes, group B, Personal work histories, 8 March 2017). Instead, rehabilitation was intended to identify those “traits” in oneself that needed to be changed:

Everyone’s work history has been discussed in turn. HP continues by asking: “Can you recognize some characteristic, something to improve on, that you ought to do something about?” One of the rehabilitees mentions “saying no” and “drawing boundaries” and another says that they are “too nice, a doormat” at the workplace. HP: “This all began with learning to say no, you remember.” [Field notes, Personal work histories, group A, 2 March 2017.]

## Discussion

In this article we have analyzed how burnout as a problem is discursively constructed, how recovery is viewed and how people dealing with burnout are categorized by HPs in the institutional context of burnout rehabilitation. The study shows that the discursive practices of rehabilitation reproduce the individual-centered discourse around burnout and there is a paucity of working life perspectives. Psychological, evolutionary, biomedical, and healthy lifestyle discourses encourage people who experience burnout to engage in an array of mind- and body-focused practices of self-care as means to work toward better management of mental distress.

Especially within the psychological and healthy lifestyle discourses, individuals were responsibilized for the problem and its solution. In the psychological discourse, burnout was constructed by pathologising specific psychological characteristics. When work-related exhaustion was considered an individual issue to be self-managed it was *the self*, in particular that was subjected to “change work” (for the concept, see Juhila et al., 2015). The healthy lifestyle discourse represents a wider discourse of “healthism” which is preoccupied with “personal health as a primary—often the primary—focus for the definition and achievement of wellbeing”; a goal which is to be attained primarily through the modification of lifestyles, with or without therapeutic help” (Crawford, 1980: 368). Healthy lifestyles have historically been the primary target of individual-level interventions that aim at preventing disease and promoting health (Korp, 2010). From a critical point of view, the strong focus on the individual’s personal characteristics and lifestyle may further reinforce the ethos of responsibilization by constructing self-work as the solution to ill-health (Korp, 2010).

The evolutionary and biomedical discourses also construct individualized interpretations of burnout, but in addition, they address that exhausted employees are either vulnerable to the “syndrome” or at the mercy of “modern” life. When burnout as a phenomenon is traced to biological issues in a deterministic way in the evolutionary discourse (Björklund and Wright, 2017) it does not appear as a fault in the self. For rehabilitees, it can be relieving to perceive burnout as a form of vulnerability which applies to all human beings and is an understandable reaction to the pressures of working life. However, the evolutionary and biomedical discourses do not recognize that people burn out in specific work contexts.

In the biomedical discourse burnout is not traced only to intra-psychological deviances and it receives a legitimate “syndrome” status. On the one hand, receiving recognition of mental distress may be especially important for people who suffer from conditions such as burnout which do not have a legitimate diagnostic status and may, therefore, leave the individual with a sense of being mistrusted (Korhonen and Komulainen, 2018). On the other hand, when burnout becomes understood within the framework of mental disease, that is depression, problems at work are shifted to the psychological and physical level (Korhonen and Komulainen, 2018; Korhonen et al., 2020; Rikala, 2013).

The gendered categorization produced in the psychological discourse is partly explained by the fact that the participants were almost exclusively women. However, the categorization of people dealing with burnout as “good girls” represents a broader popular discussion around burnout, at least in Finland. This is based on the interpretation that excessive care for other people, the tendency to bear responsibility, as well as a lack of assertiveness make women prone to burnout (Rikala, 2013). The repertoire of self-care embedded in the psychological discourse simultaneously reproduces and challenges the cultural norm of caring femininity, embodied in the “good girl syndrome,” and legitimizes “living for the self” (see also Salmenniemi and Kemppainen, 2020). The categorization of women who experience burnout as “good girls” is a problematic view because it ignores structural gender inequalities in working life, gendered practices at the workplace as well as women’s roles and responsibilities both at work and home that contribute to their burnout (Rikala, 2013).

There was only one discourse—that of welfare—in which recovery from burnout was approached from the non-individualized point of view. It seems that the historically older, more collective ideals of the Finnish health care and rehabilitation system still echo alongside the newer ones. We suggest that the discursive practices that convey the ethos of welfare and the related ideals of solidarity, social protection and equity play an important role in highlighting the participants’ stressful work situations and may, therefore, help to legitimate their mental distress and alleviate sheer individual responsibility linked to the causes of burnout and means of recovery.

So far there has been very little discussion concerning burnout and its individualized definitions from a critical and DA point of view. There is a lack of research examining the ways by which individualization manifests itself at the grassroot level of health care and rehabilitation. We suggest that discursive and category analysis are particularly fruitful tools for revealing the normative judgments of what are acceptable and valuable ways of dealing with burnout in this context. In this respect, the study provides HPs with perspectives to critically reflect on discursive effects that arise from problem construction—that is, HPs’ power in setting the limits on what can be thought and said about burnout and what ways of thinking about a problem may be silenced (Georgaca, 2013). Moreover, HPs need to acknowledge their power in producing the subject positions and related expectations and responsibilities for clients.

This study is limited, firstly, by its focus on the Finnish context and a single type of burnout intervention. Therefore, the results gained in this study are not intended to be generalizable but rather transferable to similar contexts. Secondly, the data gathered by means of hand-written field notes imposes restrictions for detailed documentation of interaction. However, field notes proved to provide rich and legitimate data for DA purposes. Instead of analyzing fine-grained interactions, the aim of our study has been to illustrate the discursive construction of the burnout phenomenon in a particular context. Our analysis provides perspectives for further interaction-oriented analyses.

As we have demonstrated, the group discussions included moments when the participants challenged the individualized notions of burnout and recovery, although this has been beyond the scope of this study. Further research is needed to explore how the rehabilitation participants negotiate—adopt and resist—the discourses and categories offered by the HPs and whether their own perceptions change temporally during the rehabilitation. More research is also required to examine the effects of the individual-centered discourse—both on rehabilitees (how they come to regard themselves as (un)healthy subjects; what are the lived effects of specific problematizations of burnout on people’s lives) and HPs (how they see their potential to influence rehabilitees’ recovery). Finally, further research is needed to analyse how HPs perceive men’s burnout and whether gendered categories related to masculinity and burnout are constructed.

## Conclusion

Although the understanding of burnout in the context of Finnish burnout rehabilitation is individual-centered, it is not neoliberal *per se* (cf. Mueller and Morley, 2020). Rather, the discursive constructions of burnout can be explained by a long tradition of the individualization of burnout especially within clinical practice. However, individual-centered understandings provide a fertile ground for neoliberal ideals and practices which emphasize the self-management of mental distress even more strongly (Brown and Baker, 2012). According to Esposito and Perez (2014: 414), “the tendency to treat “mental illness” as a problem within the individual continues to be supported within the prevailing neoliberal logic that downplays the social realm, treats individuals as self-contained agents, and pathologises thoughts and behaviors that deviate from what the market defines as functional, productive, or desirable”. Thus, as Brown and Baker (2012: 17) put it, individuals under neoliberalism are required to reconfigure the self in such a way that emotions—particularly those that might disrupt productivity, such as stress and anxiety—are self-monitored, privatized, and constrained.

We suggest that HPs employ discourses which emphasize self-care to address individual responsibility in the name of patient-centredness and empowerment (Salmon and Hall, 2003; see also Bech Risor and Lillevoll, 2021) – the values underpinning Finnish rehabilitation. In this respect—and considering the power of psychological knowledge and discourse in medical practice (Salmon and Hall, 2003)—this sort of approach seems understandable and is probably well-intentioned. Moreover, as Juhila et al. (2016) put it, HPs as grassroots level clinicians mediate between health care policies and clients’ needs and realities; they have power over clients’ lives but at the same time, they are themselves controlled by the health authorities and institutional procedures and practices which regulate their field of activity. We suggest that treating burnout as psychological-level phenomenon individualizes clients’ work-related problems so that the HPs *can address* them when there is a lack of both resources and approaches to try to influence clients’ actual work environments.

An approach that emphasizes burned out people as self-responsible managers of their mental distress may indeed appear at first glance to be respectful of individual agency. In previous research on Finnish burnout rehabilitation individuals’ enhanced sense of self-responsibility has also been linked to the strengthening of personal agency as a desirable outcome of rehabilitation (Salminen, 2020). As Bech Risor and Lillevoll (2021: 3) point out, individuals whose distress does not meet the diagnostic criteria struggle with legitimacy of their illbeing, both in working life and in the health system, from which they hope to receive help. Some clients may eagerly engage in the idea of self-management because it gives credibility to being ill while also fulfilling the norm of being a capable and agentive person who takes control over their actions and behavior (Bech Risor and Lillevoll, 2021).

We argue, however, that the major problem of individual-centered discourses around burnout is that they ignore the problems of working life, everyday stressors at work as well as social power structures and hierarchies behind the burnout syndrome. Moreover, there is research evidence suggesting that compared to person-directed interventions, hybrid type interventions with a combination of both person- and organization-directed approaches have longer-lasting positive effects in terms of alleviating burnout symptoms (Ahola et al., 2017; Awa et al., 2010; Maslach, 2017; Salminen, 2020).

Based on this study, we argue that hybrid type interventions that attempt to influence both individual- and work-related problems behind burnout would also help to prevent people dealing with burnout from being over-responsibilized for solving problems at the workplace. However, the implementation of such interventions requires financial resources and is, therefore, not just in the hands of HPs but a matter of political priorities and decisions.

## Notes

1. Burnout is a syndrome characterized by high levels of exhaustion, negative attitudes toward work (cynicism) and reduced professional efficacy (Maslach et al., 2001). Burnout is not included in the Diagnostic and Statistical Manual of Mental Disorders (DSM-5), and only appears as an additional diagnosis (Z 73.0) in the International Statistical Classification of Diseases and Related Health Problems (ICD-11).

2. We acknowledge that the debate within the half-century tradition of burnout research is extensive and diverse. Here we bring out the perspectives that are relevant to our research design.

3. The terms “occupational” and “professional” burnout have also been used in scientific research. In English the term “burnout” is frequently used when taking about burnout as a job-related phenomenon. In Finnish, the term is “työuupumus” which, by a definition, includes *work* (*työ*).

4. However, illness or disability remain the basis and starting point for granting rehabilitation, and the aim is to maintain and improve the working ability.

5. Työuupumuskuntoutuskurssi in Finnish.

6. No statistics are available on the gender distribution, educational background, or occupations of those who have participated in Kela’s burnout rehabilitation courses. The gender distribution of our data is presumably random. Burnout rehabilitation courses have been either mixed groups of different professionals (such as in our case) or targeted at a specific professional group.

7. The researcher recorded only brief notes about activities, impressions, emotional tones, and other nuances of the situation.

8. Työuupumuksen itsehoito in Finnish.

9. Direct quotations of the speakers are in quotation marks. Other parts of the data examples have been originally written down in the field notes but later edited, if needed, by using filler sentences and by picking up excerpts from different parts of the same group discussion, in order to make the core of the discursive practice visible. Data examples have been translated from Finnish to English by a professional translator.
